# Supplementary material for: Chromothripsis during telomere crisis is independent of NHEJ, and consistent with a replicative origin
Source: Genome Res. 2019 May;29(5):737–49. doi: 10.1101/gr.240705.118 (PMC6499312; doi:10.1101/gr.240705.118)
Supplement: Supplemental Material [file supp_gr.240705.118_Supplemental_file_1.zip › contigs/annotated_contigs/DB73/contig.2.DB73_length_527_mean_cov_12.4667931689.docx]

**DB73_length_527_mean_cov_12.4667931689**

```
ATGACGTATCTTCTTGTATTTGCTTCCAGGGAATCATGCTTGTTGGTGATGGATACAGAGGAAAAACTTCACCCCATACACCCAATGAA
 >chr3:189691475-189691822 - E=3e-176 p=0e+00
AAGTTTGAAGGTGCAACTGTCCTGAAAGCACTCAAAGTAAAGTCTTAACTTCTTTTGGGTTTGAGTTCCTTAGTGTCAGCCTAAGGCTT

CTGTAAATGGCAGATATAAGAAAGAAAAAAGTGGCCAGGCACAGTGGCTCACGCCTGTAATCCCAGCACTTTGGGAGGCCGAGGCGGGC

GGATCACGAGGTCGGGAGTTCGAGACCATCCTGGCTAACACGGTGAAACCCCGTCTCTACTAAAAATACAAAAAAATTAG|TTGAGTGT

GGTGGGCACA|TGTAATCCCAGCTACTCGGGAGGCTGAGGTAGGAGAACCACTTGAACCTGGGAGGTGGAGGTTACAGTGAGTCAAGAT
           >chr7:2897650-2897808 + E=2e-42
TGCACCATTGCACTCCAGCCTGGGCAACAAGAGGGAAACTCCGTCTCAAAAAAAAAAGAAAAAAAGAAAGAAAAGAAAA|GCAAA
```
